# QTL mapping of key phenological and morphological traits in grain amaranth (*Amaranthus hypochondriacus* L.)

**DOI:** 10.1270/jsbbs.25032

**Published:** 2025-11-14

**Authors:** Ahmad Zaelani, Sachiko Isobe, Kenta Shirasawa, Yosuke Yoshioka

**Affiliations:** 1 Graduate School of Science and Technology, University of Tsukuba, Tsukuba, Ibaraki 305-8572, Japan; 2 Kazusa DNA Research Institute, Kisarazu, Chiba 292-0818, Japan; 3 Institute of Life and Environmental Sciences, University of Tsukuba, Tsukuba, Ibaraki 305-8572, Japan

**Keywords:** anthesis, plant height, betalain, ddRAD-seq, grain amaranth, QTL mapping

## Abstract

Grain amaranth is a promising alternative food grain owing to its nutrient-rich composition and ability to grow under poor environmental conditions. In this study, we aimed to identify quantitative trait loci (QTLs) associated with key morphological and phenological traits using an F_2_ population of *Amaranthus hypochondriacus* derived from two commercial cultivars, Golden and Pygmy Torch. Nine traits were evaluated across three cultivation trials, and single nucleotide polymorphism (SNP) genotyping conducted using double digest restriction site-associated DNA sequencing analysis. Genetic linkage maps were constructed based on SNP markers, and QTL analysis independently conducted for each trial. Three linkage maps were constructed, spanning 5640, 5970.3, and 6960.2 cM, respectively. In total, 12 QTLs were associated with the nine traits detected on Chr. 4, 6, 9, 10, and 16 in at least two cultivations, with QTL regions of the five traits overlapping on Chr. 4. Notably, seven QTLs (*AhPH6*, *AhSTD4*, *AhNN4*, *AhBI4*, *AhHC16*, *AhIFC16*, and *AhSCC9*) related to important morphological traits were consistently detected in all three cultivation trials. The study results provide valuable genetic insights for the improvement of *A. hypochondriacus* and suggest the possibility of using molecular markers for the selection of important traits to establish breeding programs more efficiently.

## Introduction

Grain amaranth (*Amaranthus hypochondriacus* L.) is a promising crop with potential as an alternative to major food grains. Three major species of grain amaranth exist: *A. hypochondriacus*, *A. caudatus*, and *A. cruentus*. The *A. caudatus* species originates from South America, whereas *A. cruentus* and *A. hypochondriacus* originate from Central and North America, respectively (Joshi *et al.* 2018). Each species has traits identified by typical branching patterns, plant height, main inflorescence size, flowering time, and days to maturity (Espitia 1994). The cultivation of grain amaranths has a long history in their places of origin and South Asia, with many landraces and modern cultivars developed in the 1970s and 1980s. Grain amaranths are not widespread in Japan but are produced in some areas, such as the Iwate, Akita, and Nagano prefectures (JSAPR 2025). However, research on the genetics and molecular breeding of amaranth is not as advanced as that of other major crops. Grain amaranths have gained worldwide attention as a new functional food and are known for their abundant nutrient-rich seeds (Barba de la Rosa *et al.* 2009, Caselato-Sousa and Amaya-Farfán 2012). In addition, they are highly adaptable to abiotic and biotic stressors (Anuradha *et al.* 2023, Netshimbupfe *et al.* 2023, Pulvento *et al.* 2022). Thus, the improvement of grain amaranth has the potential to address global food security challenges, such as climate change and growth.

Despite being categorized as a prospective crop, grain amaranth remains underexplored, and genetic studies are limited. The first genetic linkage map of grain amaranth was constructed for the *A. cruentus* F_2_ population using microsatellite markers (Mallory *et al.* 2008). Later, a more detailed linkage map employing single nucleotide polymorphism (SNP) markers was developed for the interspecific F_2_ population of *A. hypochondriacus* × *A. caudatus* (Maughan *et al.* 2011). Some studies have focused on color-related traits in *A. hypochondriacus*, including seed coat, stem, leaf, and inflorescence color (Lightfoot *et al.* 2017, Stetter *et al.* 2020, Winkler *et al.* 2024). Lin *et al.* (2022) performed an interspecific genome-wide association study and detected several candidate genes for days to flowering in grain amaranths. In *A. cruentus*, a locus responsible for seed shattering has been identified (Kondo *et al.* 2024). Furthermore, high-quality reference genomes of grain amaranths have been published (Clouse *et al.* 2016, Lightfoot *et al.* 2017, Ma *et al.* 2021, Sunil *et al.* 2014, Winkler *et al.* 2024), providing valuable resources to facilitate the genetic analyses of important traits of interest in grain amaranth breeding programs.

Although past grain amaranth breeding has led to improvements in various traits, further improvements are needed in traits such as seed size, early growth, shedding habits, lodging resistance, harvest suitability, seed quality, and high protein content (Das 2016). Many of these breeding issues remain, and their resolution is necessary to further popularize grain amaranth worldwide. Many amaranth cultivars have been developed for ornamental purposes, some of which have unique traits that may be useful for grain-type cultivars. Some ornamental cultivars of *A. hypochondriacus* are well known for their dwarf and early mature plants. These cultivars possess valuable genes involved in plant architecture and phenology. Transferring beneficial traits to grain cultivars can improve lodging resistance, optimize planting density, and expand adaptability.

In this study, we aimed to investigate the heritability of important traits of *A. hypochondriacus*, including dwarfness and early maturity, which are unique to ornamental cultivars, and to detect the quantitative trait loci (QTLs) involved in these traits to establish a DNA marker-based selection system. To this end, we produced progenies by crossing ornamental and grain-type cultivars and evaluated the phenotypic variations in phenological and morphological traits. Genetic linkage maps were constructed using SNPs detected via double digest restriction site-associated DNA sequencing (ddRAD-seq) analysis, and QTL analysis subsequently performed. The findings of the present study provide information on the genetic foundation of marker-assisted selection in *A. hypochondriacus* genetic improvement programs.

## Materials and Methods

### Plant materials

**Fig. 1.**
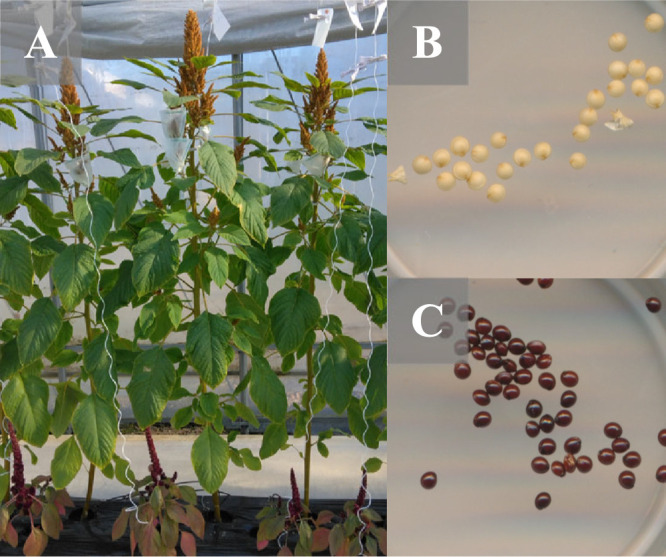
Differences in plant and seed morphology between the parental lines. A: Golden (back) and Pygmy Torch (front) plants; B: Golden seeds; C: Pygmy Torch seeds.

Two commercial cultivars of *A. hypochondriacus*, Golden (female parent) and Pygmy Torch (male parent), were selected as parents to develop the F_1_ and F_2_ populations (Fig. 1). Golden is a grain-type cultivar with tall growth, poorly developed lateral branches, and green hypocotyls. Pygmy Torch is an ornamental cultivar with a dwarf morph, fully elongated lateral branches, and red hypocotyls. After selfing the cultivars two or three times, they were crossed to obtain F_1_ seeds. The morphological marker, red hypocotyls, was used to verify the successful cross in the F_1_ generation, and F_2_ seeds produced via self-pollinating the F_1_ generation (Golden ♀ × Pygmy Torch ♂).

### Growth condition and phenotypic evaluation

The F_2_ populations with their parents and F_1_s were cultivated three times: summer 2023 (July–October 2023), autumn 2023 (October 2023–February 2024), and spring 2024 (May–July 2024). Seeds were planted in plastic pots with a diameter of 6 cm containing moist culture soil (Nihon Hiryo Co., Ltd., Fujioka, Japan). After two weeks, the seedlings were moved to 5.1-L slit pots containing peat moss (Hokkaido Peat Moss Inc., Konosu, Japan) and cultivated in a plastic house at the Tsukuba-Plant Innovation Research Center, University of Tsukuba (Tsukuba, Japan). Nine agronomic traits were characterized according to the UPOV (2008): heading date (HD), anthesis date (AD), plant height (PH), stem diameter (STD), number of nodes (NN), branching index (BI), hypocotyl color (HC), inflorescence color (IFC), and seed coat color (SCC) (Supplemental Table 1).

### ddRAD-seq analysis

DNA was extracted from the F_2_ populations and parental lines using an automated DNA extraction platform, oKtopure with the LGS sbeadex mini plant kit (LGC Biosearch Technologies, Teddington, UK). DNA quality and concentration were assessed using a spectrophotometer (NanoDrop 8000, Thermo Fisher Scientific, Waltham, MA, USA). Sequencing libraries were prepared as described by Shirasawa *et al.* (2016). Total genomic DNA was digested using the restriction enzymes, PstI and MspI. The library sequences were determined on a DNBSEQ-G400RS platform (MGI Tech, Shenzhen, China) to obtain 100-bp paired-end reads. Reads were classified by tag using SplitBarcode v.2.0.0 (MGI Tech).

### SNP calling analysis

SNPs obtained from the ddRAD-seq analysis were analyzed using the Galaxy platform (The Galaxy Community *et al.* 2024). Raw reads were quality-filtered using PRINSEQ with the following parameters: *-trim_right 1 -trim_qual_right 10 -trim_qual_type min -trim_qual_rule 1 -trim_qual_window 1 -trim_qual_step 1* (Schmieder and Edwards 2011). Adapter sequences were removed using Cutadapt (Martin 2011). Clean reads were mapped to the *A. hypochondriacus* reference genome v.2.2 (Winkler *et al.* 2024) using BWA-MEM and then sorted in coordinate order using SortSam. Finally, the Stacks pipeline for genetic mapping (*ref_map.pl*; Catchen *et al.* 2013) was used for SNP calling.

### Genetic linkage map construction

Genetic linkage maps were constructed based on SNP markers of the F_2_ populations using R/ASMap (Taylor and Butler 2017) and R/qtl (Arends *et al.* 2010). The SNPs were filtered based on the following criteria: individuals with up to 30% missing data were included; markers were required to be present in at least 90% of the population; and loci had to segregate in a 1:2:1 ratio (α = 0.05). SNPs with high genotype error logarithm of odds (LOD) scores were treated as missing data. The minimum-spanning-tree algorithm was applied to cluster and order SNP markers using the *mstmap* function (Wu *et al.* 2008). Genetic distance was calculated using the Kosambi mapping function. Linkage maps were created and visualized using R/LinkageMapView (Ouellette *et al.* 2018).

### QTL analysis

QTL analysis was performed using R/qtl (Arends *et al.* 2010). Five traits—HD, AD, PH, STD, and NN—were analyzed using the composite interval mapping method with the *cim* function (Zeng 1994). In addition, nonparametric interval mapping under a single-QTL model using the *scanone* function was performed for BI, HC, IFC, and SCC (Kruglyak *et al.* 1996). The threshold LOD score for significant QTLs was determined using 1000 permutations at *P* < 0.05. The QTL effect and proportion of variance explained (PVE) were calculated using the *fitqtl* function (method = “*hk*”). To investigate the potential interactions between QTLs, a two-dimensional genome scan using the *scantwo* function (method = “*hk*”) was performed. The LOD scores for additive, interaction, and full models were determined with 1000 permutations (α = 0.05).

### Validation of QTLs on Chr. 4 and development of markers

A total of 135 F_3_ individuals derived from eight F_2:3_ families, selected based on the genotype of the F_2_ generation, were cultivated and genotyped to validate the effects of major QTLs related to phenological and morphological traits. The F_2:3_ families included four heterozygous (H) families (30 individuals per family), two families homozygous for the female parent (Golden) allele (five individuals per family), and one family homozygous for the male parent (Pygmy Torch) allele (five individuals). Six agronomic traits that overlapped with the QTL region on Chr. 4 were evaluated: HD, AD, PH, STD, NN, and BI.

Melt curve genotyping analysis was conducted using a QuantStudio 1 Real-Time PCR System (Applied Biosystem, Waltham, MA, USA) with the MeltDoctor HRM Master Mix (Thermo Fisher Scientific). HRM markers were designed using Primer3Plus (https://primer3plus.com/) based on the QTLs on Chr. 4 (Supplemental Table 2). A 10-μL reaction was prepared following the manufacturer’s recommendation (Applied Biosystems 2009). The gDNA isolated from parents and the F_1_ generation were used as controls. The protocol was as follows: pre-denaturation at 95°C for 10 min, followed by 40 cycles of denaturation at 95°C for 15 s, and extension at 60°C for 1 min. The melting temperature was evaluated from 60–95°C with a continuous ramp mode of 0.025°C/s. The F_3_ genotype was determined by comparing the melting curve images with those of the controls. The marker-trait association was calculated using one-way ANOVA.

## Results

### Phenotypic evaluation

**Fig. 2.**
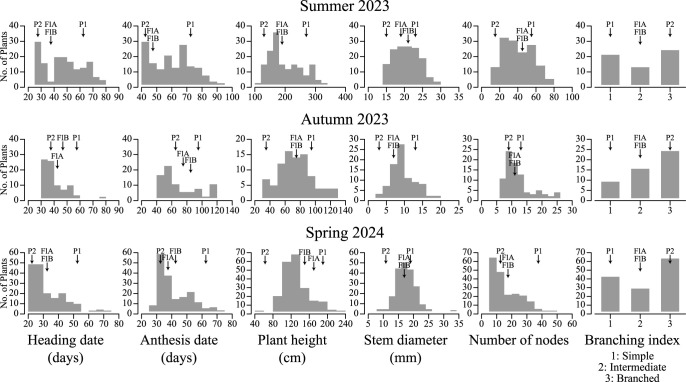
Phenotypic distribution of the F_2_ population produced through self-pollinating the F_1_ population (Golden ♀ × Pygmy Torch ♂) in summer 2023 (n = 140), autumn 2023 (n = 80), and spring 2024 (n = 189). P1: Golden; P2: Pygmy Torch; F1A: F_1_ population (Golden ♀ × Pygmy Torch ♂); F1B: F_1_ population (Pygmy Torch ♀ × Golden ♂).

Phenotypes of the nine observed traits differed among the parental lines and their F_1_ and F_2_ populations. As expected, the female parents exhibited higher PH, STD, and NN, whereas the male parents exhibited earlier flowering, as indicated by the HD and AD. In the F_1_ generation, all individuals exhibited uniform phenotypic traits. Some traits, including HD, AD, PH, STD, NN, and BI, showed intermediate values between the parents, suggesting incomplete dominance or additive genetic effects. In contrast, red hypocotyls, red inflorescences, and dark seed coats were consistently observed, indicating the dominance of these color traits. Phenotypic variations in the F_2_ population and their parental lines in the three cultivations are presented in Fig. 2 and Table 1. PH, STD, and NN showed a normal distribution, whereas HD and AD distributions were skewed toward the Pygmy Torch. The Chi-square test showed that segregation ratios of the three color-related traits generally fit the expected 3:1 Mendelian ratio with HC (*P* = 0.56, 1.0, and 0.43) and IFC (*P* = 0.53, 1.0, 0.10) in three cultivations, whereas SCC differed significantly from the expected ratio in the summer cultivation (*P* = 0.02) but fit the expected ratio in the other two cultivations (*P* = 0.23 and 0.41).

### Construction of genetic linkage maps

Three linkage maps were constructed separately for the three cultivation trials based on SNP markers in the F_2_ populations obtained from the ddRAD-seq analysis. The SNPs were filtered to select desired SNPs based on these criteria. The result identified 3,692, 3,478, and 4,234 markers, which were used to construct the genetic linkage maps (Supplemental Table 3). Each linkage map consisted of 16 linkage groups spanning 5,640, 5,970, and 6,960 cM, with average marker distances of 1.5, 1.7, and 1.7 cM, respectively.

### Identification of QTLs for the observed traits

QTL analyses were conducted independently for each trait. Of the 15 QTLs identified, 12 were detected in at least two cultivation trials with a PVE >10%, except for *AhHD10*, *AhPH6*, and *AhNN10* in summer 2023 (Supplemental Table 4). The LOD score ranged from 3.96 for *AhBI4* to 42.78 for *AhNN4*. These QTLs were distributed on Chr. 4, 6, 9, 10, and 16. Two QTLs for HD were identified on Chr. 4 (*AhHD4*) and Chr. 10 (*AhHD10*), for PH on Chr. 4 (*AhPH4*) and Chr. 6 (*AhPH6*), and for NN on Chr. 4 (*AhNN4*) and Chr. 10 (*AhNN10*). Additionally, one QTL each was detected for AD (*AhAD4*), STD (*AhSTD4*), BI (*AhBI4*), HC (*AhHC16*), IFC (*AhIFC16*), and SCC (*AhSCC9*). Seven QTLs were consistently identified in the three cultivations: *AhPH6*, *AhSTD4*, *AhNN4*, *AhBI4*, *AhHC16*, *AhIFC16*, and *AhSCC9* (Table 2). Notably, QTLs for traits such as HD, PH, STD, NN, and BI overlapped in the region on Chr. 4. A two-dimensional genome scan revealed significant additive QTLs for HD, PH, and NN on Chr. 4, 6, and 10, respectively; no statistically significant locus–locus interactions were detected except for NN in spring 2024.

### Validation of the effect that QTLs on Chr. 4 have on morphological traits

**Fig. 3.**
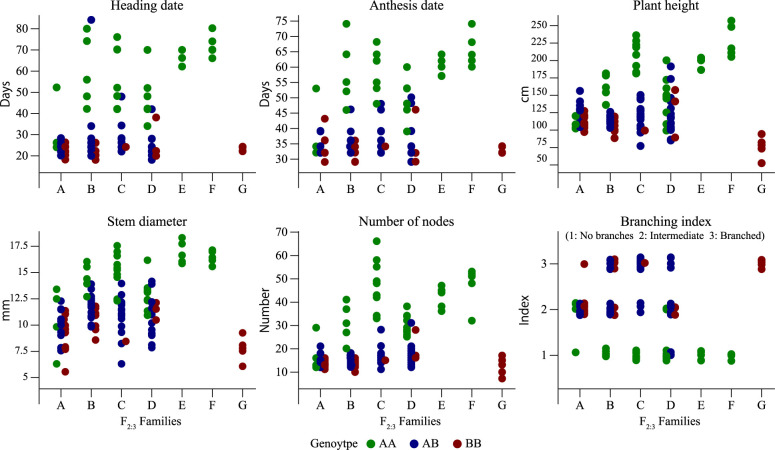
Phenotypic distributions in each F_2:3_ family and genotypes at the major QTL on Chr. 4 for each F_3_ individual. A–D: F_3_ families (n = 30 per family) derived from F_2_ individuals with the heterozygous genotype (AB). E–F: F_3_ families (n = 5 per family) derived from F_2_ individuals homozygous for the female parent (Golden) allele (AA). G: F_3_ family (n = 5) derived from an F_2_ individual homozygous for the male parent (Pygmy Torch) allele (BB).

The phenotypic distribution of F_3_ individuals revealed distinct segregation patterns among the different genotypic groups (Fig. 3). As expected, the F_2:3_ population derived from heterozygous (H) F_2_ individuals exhibited phenotypic segregation, confirming the presence of a causal gene at the target locus. In contrast, the F_2:3_ populations derived from homozygous F_2_ individuals remained phenotypically stable, consistent with their fixed allelic composition at the target locus. This observation supports the QTL effects identified on Chr. 4, as phenotypic differences among the three genotypic groups were maintained. Furthermore, an HRM marker developed from a SNP on Chr. 4 showed significant associations with the five traits: HD, AD, PH, STD, and NN (Supplemental Table 5).

## Discussion

As with other crops, the genetic improvement of *A. hypochondriacus* requires a strategic approach that integrates beneficial traits from different cultivars or genetically distant genetic resources. Several studies have identified superior grain amaranth accessions that have the potential to enhance key agronomic traits, improve yield, and increase adaptability across diverse growing conditions (Blair *et al.* 2023, Boro *et al.* 2024, Schafleitner *et al.* 2022). In the present study, a grain-type cultivar with short lateral branches, upright growth, and white seeds was crossed with an ornamental cultivar with early flowering and a semi-dwarf stature, and their progenies used for genetic analysis. The results revealed information on the inheritance of these traits, and we successfully identified several important QTLs.

The semi-dwarf and early maturity observed in the ornamental cultivars used in this study, such as Pygmy Torch, are particularly desirable for breeding compact, high-density cultivars with short crop cycles. The introduction of these beneficial traits into grain-type cultivars facilitates flexible crop rotation and enhances their adaptability to diverse environmental conditions. In the present study, QTLs associated with plant architecture and flowering time were consistently distributed across Chr. 4, 6, and 10 (Table 2, Supplemental Fig. 1). The QTL on Chr. 4 has been a focal point because of the significant effect it has on traits that influence plant architecture and yield potential, including early HD, AD, PH, STD, NN, and branching patterns. In addition, the QTL on Chr. 6 was strongly associated with PH, a trait crucial for developing cultivars with shorter stature, thereby enhancing lodging resistance and improving suitability for mechanical harvesting. Furthermore, the QTL identified on Chr. 10 influences the HD and NN on the main stem, contributing to the development of new cultivars with shorter crop cycles and lodging resistance.

A previous study reported seven genomic regions for days to flowering in grain amaranths (Lin *et al.* 2022). The two QTLs on Chr. 4 and 10 for HD and AD identified in this study were detected in regions near those reported in the previous study, suggesting that polymorphisms in these regions would play an important role for these traits in *A. hypochondriacus*. However, these QTLs were only detected in two of the three cultivation trials, and no significant QTL detected for AD in autumn 2023. This suggests that differences in environmental conditions, such as photoperiod and temperature, may have influenced gene expression in these regions. As this study did not repeat the cultivation for each season, the relationship between environmental conditions and QTLs needs to be re-examined in future studies.

The QTL regions on Chr. 4 for several traits overlapped (Table 2). This co-localization indicates the presence of pleiotropic effects or closely linked genes for these traits, which may cause selection trade-offs. A potential pleiotropic effect or strong linkage implies that selection for one trait may positively influence the selection for another. For example, selecting for shorter PH may automatically lead to early maturity in progenies derived from Pygmy Torch, although optimizing PH and flowering time independently could be challenging.

While pleiotropy can be advantageous when linked traits are beneficial, it may also present challenges if an undesirable trait is inherited along with a trait of interest (Reinert 2022). We identified a relationship between PH and branching patterns, and our results indicated that a decrease in PH was associated with an increase in lateral branching (Supplemental Fig. 2). However, the correlation between PH and STD requires further investigation. In general, semi-dwarfs are thought to contribute to lodging resistance; however, when accompanied by a decrease in STD, their contribution to lodging resistance may be limited. Therefore, careful evaluation of potential trade-offs to ensure that improvements in PH do not compromise overall plant performance or agronomic suitability is crucial. This pleiotropic effect has been reported to be responsible for the abnormal growth of dwarf plants in grain amaranth (Kulakow 1987).

Our results revealed a single QTL for HC and IFC located on Chr. 16, whereas seed color was associated with a distinct QTL on Chr. 9, consistent with the findings of previous studies (Stetter *et al.* 2020, Winkler *et al.* 2024). This finding confirms that these traits follow a simple inheritance pattern, as previously reported by Gupta and Gudu (1990). The independent regulation of seed color suggests distinct biosynthetic pathways or genetic control mechanisms, reducing the risk of unintended correlations with other agronomic traits. Genetic independence is particularly advantageous because it allows breeders to selectively target seed color without inadvertently influencing plant growth or flowering characteristics.

In the present study, we successfully identified QTLs associated with important agronomic traits in *A. hypochondriacus* that could contribute to the genetic improvement of plant types and early flowering traits. Fine mapping of the detected QTLs and validation of candidate genes are essential for subsequent research (Schaid *et al.* 2018). Additionally, multi-environment trials are necessary to confirm the stability of the identified loci and ensure their applicability in breeding programs. The findings of this study provide valuable genetic insights useful for the improvement of *A. hypochondriacus* using targeted breeding strategies. Furthermore, the study results suggest the possibility of using molecular markers to select important traits to establish breeding programs more efficiently. The ability to develop semi-dwarf, early maturing cultivars with a preferred seed color expands the potential for widespread grain amaranth production worldwide, including in Japan.

## Author Contribution Statement

Y.Y. conceptualized and supervised the study. A.Z. conducted the experiments. S.I. and K.S. performed the ddRAD-seq analysis. A.Z. and Y.Y. wrote, reviewed, and edited the manuscript. All authors read and approved the final article.

## Supplementary Material

Supplemental Figures

Supplemental Tables

## Figures and Tables

**Table 1.** Segregation of color in the hypocotyls, inflorescences, and seed coats of the parental lines, their reciprocal F_1_s, and F_2_ in three cultivation trials

	HC			IFC			SCC	
	Red	Green		Red	Green		Dark	White
Summer 2023								
P1		3			3			3
P2	3			3			3	
F1A	4			4			4	
F1B	4			4			4	
F_2_	102	38		101	38		92	47
Autumn 2023								
P1		4			4			4
P2	3			3			3	
F1A	3			3			3	
F1B	2			2			2	
F_2_	60	20		60	20		26	13
Spring 2024								
P1		4			4			4
P2	4			4			4	
F1A	3			3			3	
F1B	4			4			4	
F_2_	137	52		116	51		92	36

P1: Golden; P2: Pygmy Torch; F1A: F_1_ (Golden ♀ × Pygmy Torch ♂); F1B: F_1_ (Pygmy Torch ♀ × Golden ♂); F_2_: F_2_ (selfing of F1A) HC, hypocotyl color; IFC, inflorescence color; SCC, seed coat color

**Table 2.** Summary of the quantitative trait loci for nine agronomic traits commonly detected in more than two cultivation trials

Trait	Abbreviation	QTL ID	Chr.	Estimated range*^a^*	Cultivation trial*^b^*
Heading date	HD	*AhHD4*	4	15,620,178–19,903,179	A2023, S2024
		*AhHD10*	10	14,674,403–15,504,864	S2023, S2024
Anthesis date	AD	*AhAD4*	4	15,620,178–22,320,068	S2023, S2024
Plant height	PH	*AhPH4*	4	18,144,314–21,858,471	S2023, S2024
		*AhPH6*	6	13,941,137–20,369,330	S2023, A2023, S2024
Stem diameter	STD	*AhSTD4*	4	18,218,753–22,320,068	S2023, A2023, S2024
Number of nodes	NN	*AhNN4*	4	17,450,913–22,320,068	S2023, A2023, S2024
		*AhNN10*	10	14,674,403–15,675,389	S2023, S2024
Branching index	BI	*AhBI4*	4	10,968,016–22,643,818	S2023, A2023, S2024
Hypocotyl color	HC	*AhHC16*	16	5,048,763–6,076,285	S2023, A2023, S2024
Inflorescence color	IFC	*AhIFC16*	16	5,048,763–6,076,285	S2023, A2023, S2024
Seed coat color	SCC	*AhSCC16*	9	14,135,980–17,335,905	S2023, A2023, S2024

*^a^* Estimated range within the *Amaranthus hypochondriacus* v.2.2 genome *^b^* A2003: Autumn 2023; S2023: Summer 2023; S2024: Spring 2024
