# Supplementary material for: QTL mapping of key phenological and morphological traits in grain amaranth (Amaranthus hypochondriacus L.)
Source: Breed Sci. 2025 Nov 14;75(5):392–9. doi: 10.1270/jsbbs.25032 (PMC13129576; doi:10.1270/jsbbs.25032)
Supplement: Supplementary file 1 — Supplemental Figures [file 75_392_s1.pdf]

Zaelani et al./Breeding Science
doi: 10.1270/jsbbs.25032

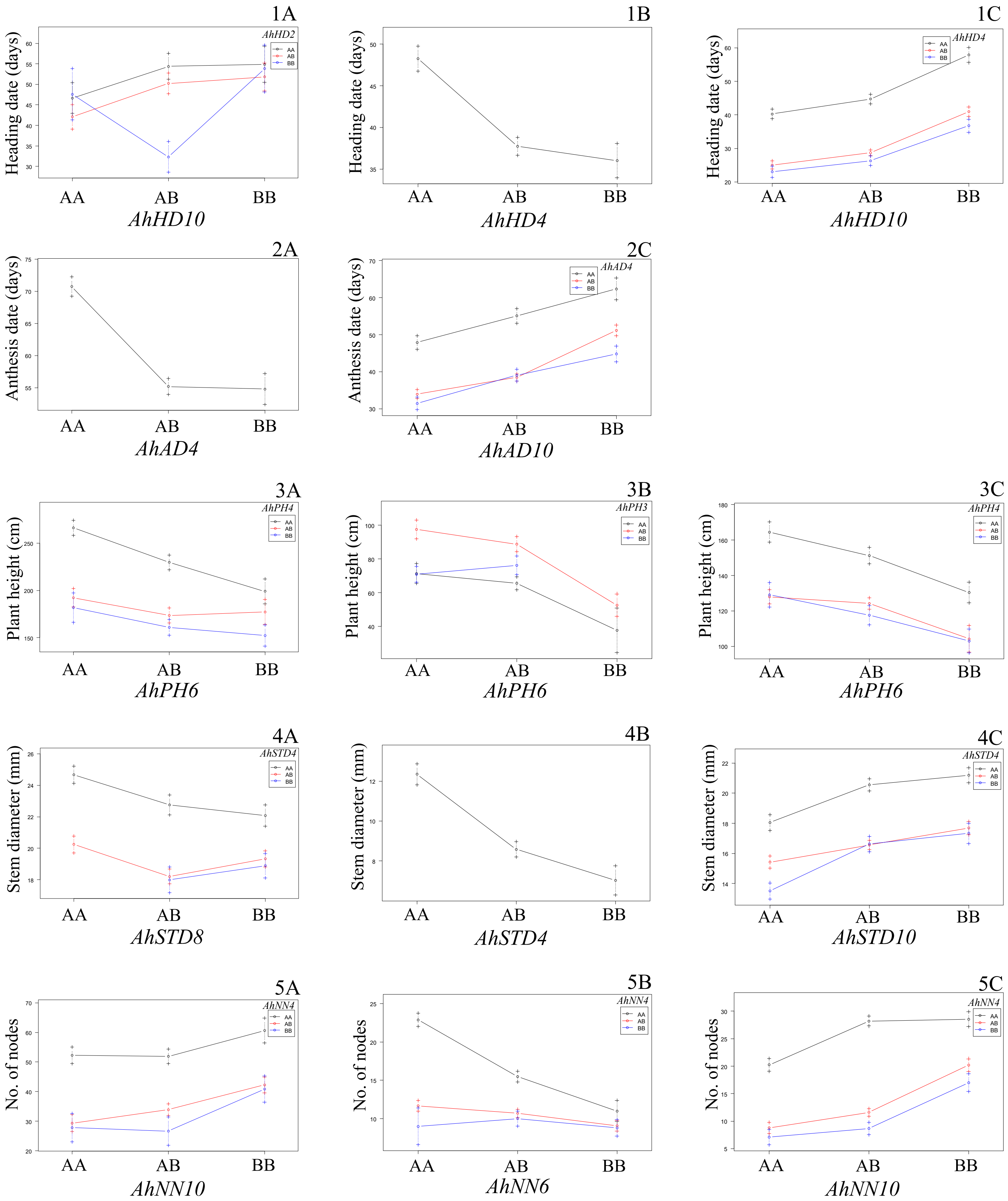

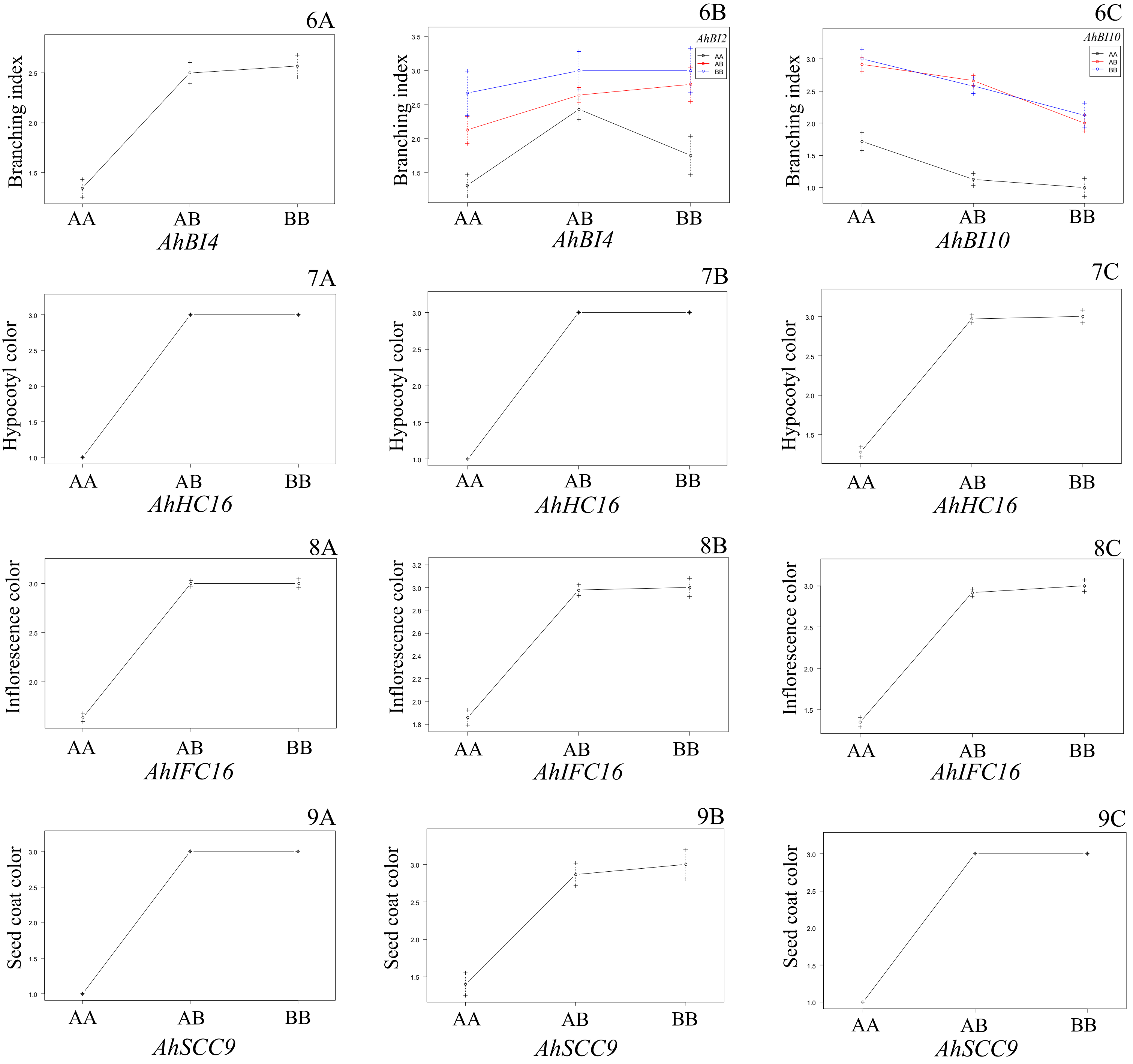

Supplemental Fig. 1. QTL effect plots on nine traits, the y-axis represents the phenotype and the x-axis respresents the marker genotypes. (1). heading date, (2) anthesis date,(3). plant height, (4). stem diameter, (5). no. of nodes, (6). branching index, (7).hypocotyl color, (8). inflorescence color, and (9). seed coat color, across three cultivations (A). summer 2023, (B). autumn 2023, (C). spring 2024.

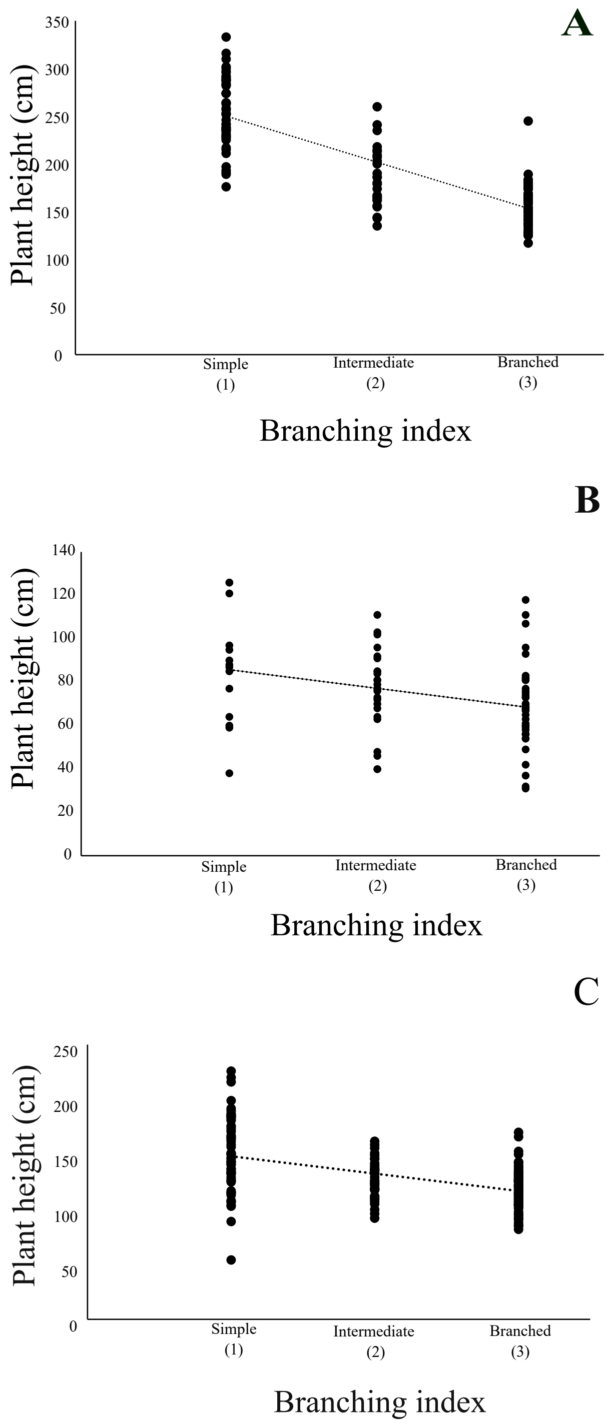

Supplemental Fig. 2. Relationship between plant height and branching index in summer 2023 (**A**), autumn 2023 (**B**), and spring 2024 (**C**)
