# Supplementary material for: QTL mapping of key phenological and morphological traits in grain amaranth (Amaranthus hypochondriacus L.)
Source: Breed Sci. 2025 Nov 14;75(5):392–9. doi: 10.1270/jsbbs.25032 (PMC13129576; doi:10.1270/jsbbs.25032)
Supplement: Supplementary file 2 — Supplemental Tables [file 75_392_s2.pdf]

doi: 10.1270/jsbbs.25032

Supplemental Table 1. Description of nine traits observed in this study

**Trait**	**Abbreviation**	**Description**	**Data-type**	**Category**
Heading date (days)	HD	Measurement of the days to heading from sowing date	Numerical	-
Anthesis date (days)	AD	Measurement of the days to anthesis from sowing date	Numerical	-
Plant height (cm)	PH	Measurement of the height of a plant from the base to the top of main inflorescence	Numerical	-
Stem diameter (mm)	STD	Measurement of the diameter of the main stem at ±10 cm from the base	Numerical	-
Number of nodes (no.)	NN	Measurement of nodes were counted along the main stem	Numerical	-
Branching Index	BI	Visual observation	Categorical	(1). Simple: no lateral branch or a few lateral branches with short length; (2). Intermediate: Erect, more lateral branches with medium length; (3). Branched: Many long lateral branches and/or main inflorescence unidentified
Hypocotyl color	HC	Visual observation	Categorical	(0) Green; (1) Red
Inflorescence color	IFC	Visual observation	Categorical	(0) Green; (1) Red
Seed coat color	SCC	Visual observation	Categorical	(0) White; (1) Dark

Supplemental Table 2. High-resolution melting (HRM) Primer information

**Primer ID**	**SNP**	**Chr**	**Primer Sequence**	**SNP Physical location based on *A. hypochondriacus* v2.2 genome**
AH002	A/T	4	Forward: 5' ACACCTCCAAAGCGGTTAGG 3'<br>Reverse: 5' TGCAGAACCTCCTCTGTGTG 3'	19,967,495

Supplemental Table 3. Distribution of SNP markers on 16 linkage groups from three linkage maps

Linkage groups (LGs)	No of markers			Total Length (cM)			Average of Interval (cM)			Max. of Interval (cM)		
	**1st**	**2nd**	**3rd**	**1st**	**2nd**	**3rd**	**1st**	**2nd**	**3rd**	**1st**	**2nd**	**3rd**
1	351	390	436	550.7	730	622.6	1.6	1.9	1.4	18.6	21.4	13.1
2	456	332	430	871.2	578.7	840.8	1.9	1.7	2	26.7	16.9	12.8
3	343	282	330	563.9	516.7	489.3	1.6	1.8	1.5	12	17.3	10.7
4	149	211	237	181.7	331.5	280.7	1.2	1.6	1.2	15.3	11.5	12.9
5	270	235	256	494	459.7	716.7	1.8	2	2.8	14.3	13.2	19.1
6	286	239	300	383.9	370.1	531	1.3	1.6	1.8	10.8	12.8	12.5
7	220	188	225	253.6	397.4	353.7	1.2	2.1	1.6	9.9	13.7	11.1
8	243	238	266	348.1	382.4	409.4	1.4	1.6	1.5	11.1	16.9	17.4
9	244	172	258	347.6	268.6	408.8	1.4	1.6	1.6	11.5	11.9	15.4
10	225	190	232	343.1	364.6	370.6	1.5	1.9	1.6	12.1	15.3	10.3
11	267	241	266	300	378.3	304.1	1.1	1.6	1.1	11.5	21	14.2
12	68	87	235	219.5	161.2	446.2	3.3	1.9	1.9	21.3	12.2	14.3
13	216	215	258	321.7	366.4	426.6	1.5	1.7	1.7	12.2	16.9	17.5
14	197	173	200	212.1	286.6	324.1	1.1	1.7	1.6	11.3	19.6	11.9
15	58	171	193	54.7	225.6	231.4	1	1.3	1.2	7.9	13.4	11.7
16	99	114	112	194.2	152.6	204.1	2	1.4	1.8	11.7	7.8	10.4
**Overall**	**3692**	**3478**	**4234**	**5640**	**5970**	**6960**	**1.5**	**1.7**	**1.7**	**26.7**	**21.4**	**19.1**

1st : Summer 2023

2nd : Autumn 2023

3rd : Spring 2024

Supplemental Table 4. The QTLs information detected in three cultivations

Cultivation	Trait	QTL ID	Chr	LOD	1.5 LOD support interval (bp)	PVE (%)	QTL effects	
							Additive	Dominant
Summer 2023	Heading date	*AhHD2*	2	7.72	25,738,564-26,193,097	6.86	-6.06	2.15
		AhHD10	10	12.26	14,674,403-15,504,864	4.99	4.61	-0.71
	Anthesis date	*AhAD4*	4	18.9	21,036,246-22,320,068	36.25	-8.53	-8.24
	Plant height	*AhPH4*	4	23.56	21,036,246-21,858,471	34.76	-39.19	-23.39
		AhPH6	6	7.33	20,369,330-20,738,671	8.08	-19.49	-9.66
	Stem diameter	*AhSTD4*	4	16.79	21,036,246-22,320,068	32.40	-2.14	-2.07
		AhSTD8	8	6.95	15,264,869-15,819,864	6.60	-0.86	-1.28
	Number of nodes	*AhNN4*	4	22.03	21,036,246-22,320,068	40.84	-12.16	-8.56
		AhNN10	10	7.09	14,674,403-15,326,146	8.30	5.93	-3.90
	Branching index	*AhBI4*	4	13.70	21,036,246-21,858,471	99.49	1.00	1.03
	Hypocotyl color	*AhHC16*	16	35.50	5,055,013-6,076,285	99.99	0.50	0.50
	Inflorescence color	*AhIFC16*	16	35.40	5,055,013-6,076,285	99.99	0.50	0.50
	Seed coat color	*AhSCC9*	9	30.10	14,136,143-15,053,781	100	0.50	0.50
Autumn 2023	Heading date	*AhHD4*	4	11.8	17,592,701-19,903,179	33.88	-6.25	-4.47
	Anthesis date	-	-	-	-	-	-	-
	Plant height	*AhPH3*	3	9.17	23,204,711-24,215,610	12.71	1.41	21.67
		AhPH6	6	10	13,941,137-15,375,067	15.30	-19.47	15.80
	Stem diameter	*AhSTD4*	4	12.20	18,218,753-20,153,174	38.03	-2.69	-1.15
	Number of nodes	*AhNN4*	4	23.40	18,807,424-19,903,179	43.14	-3.36	-3.12
		AhNN6	6	15.90	23,779,748-24,456,538	16.55	-2.53	-0.65
	Branching index	*AhBI2*	2	4.41	3,664,819-30,981,885	90.90	0.97	1.03
		AhBI4	4	3.96	10,968,016-22,643,818	18.63	0.48	0.04
	Hypocotyl color	*AhHC16*	16	17.60	5,120,811-6,076,285	90.52	0.48	0.50
	Inflorescence color	*AhIFC16*	16	17.60	5,120,811-6,076,285	90.52	0.48	0.50
	Seed coat color	*AhSCC9*	9	6.12	14,135,980-17,335,905	63.86	0.43	0.36
Spring 2024	Heading date	*AhHD4*	4	29.29	15,620,178-17,971,778	46.08	-9.23	-6.58
		AhHD10	10	16.70	14,674,403-15,326,146	27.48	7.96	-4.14
	Anthesis date	*AhAD4*	4	27.28	15,620,178-17,971,778	35.01	-8.37	-6.26
		AhAD10	10	22.57	14,334,191-15,399,706	31.49	7.86	-2.35
	Plant height	*AhPH4*	4	15.59	18,144,314-20,153,174	24.99	-15.97	-13.72
		AhPH6	6	7.71	18,574,484-18,733,077	10.94	-13.84	6.18
	Stem diameter	*AhSTD4*	4	26.48	18,723,787-20,153,174	35.93	-2.11	-1.44
		AhSTD10	10	9.69	14,085,555-14,308,121	14.32	1.47	0.64
	Number of nodes	*AhNN4*	4	42.78	17,450,913-17,971,778	55.30	-7.92	-5.49
		AhNN10	10	12.53	14,674,403-15,675,389	15.05	5.03	-0.84
	Branching index	*AhBI4*	4	20.79	15,620,178-20,153,174	88.41	0.91	1.05
		AhBI10	10	5.64	13,179,836-16,636,337	50.66	0.67	0.69
	Hypocotyl color	*AhHC16*	16	40.80	5,048,763-6,076,285	88.45	0.46	0.52
	Inflorescence color	*AhIFC16*	16	37.10	5,048,763-6,076,285	87.44	0.50	0.46
	Seed coat color	*AhSCC9*	9	33	14,255,476-14,774,673	100.00	0.50	0.50

Supplemental Table 5. Analysis of variance (ANOVA) marker-trait association

Trait	Df	SS	MS	F-value	*p* -value
Heading date	2	6127	3063.3	101.10	<0.001***
	128	3877	30.3		
Anthesis date	2	11091	5546	122.90	<0.001***
	128	5776	45		
Plant height	2	108941	54470	67.63	<0.001***
	128	103088	805		
Stem diameter	2	466.1	233.07	59.55	<0.001***
	128	500.9	3.91		
Number of nodes	2	12375	6188	112.90	<0.001***
	128	7013	55		

*, **, and *** indicate significance at a = 0.05, 0.01, and 0.001, respectively. n.s.=not significant
